# Diffuse leptomeningeal enhancement in neurosarcoidosis-related longitudinally extensive myelitis

**DOI:** 10.1055/s-0043-1772604

**Published:** 2023-10-13

**Authors:** Fabiano Ferreira de Abrantes, Marianna Pinheiro Moraes de Moraes, José Luiz Pedroso, Orlando G. Barsottini

**Affiliations:** 1 Universidade Federal de São Paulo, Escola Paulista de Medicina, Departamento de Neurologia e Neurocirurgia, São Paulo SP, Brazil.

**Figure 1:**
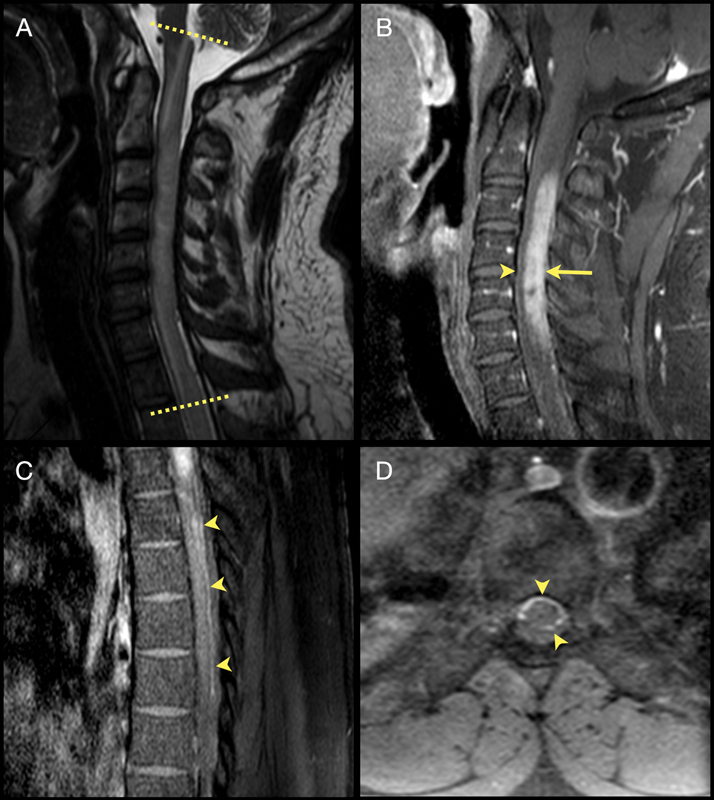
T2-weighted ( **A** ) image of the cervical spine demonstrates a longitudinally extensive myelitis (between the dashed lines). Contrast-enhanced T1-weighted ( **B** ) image with an extensive posterior spinal enhancement (arrow), and an anterior meningeal enhancement (arrowhead). Contrast-enhanced T1-weighted ( **C** ) of the thoracic spine disclosing an extensive posterior leptomeningeal enhancement (arrowheads), and ( **D** ) a circumferential involvement of the thoracic leptomeninges (arrowhead).

A 35-year-old man presented with a 1-month history of bilateral lower limb weakness and numbness. On examination, he had paraparesis with lower limbs hyperreflexia, and T5 sensory level. Magnetic resonance imaging (MRI) of the spine revealed a longitudinally extensive myelitis (LETM) with a diffuse leptomeningeal enhancement ( Figure 1 ). Thorax computed tomography (CT) disclosed bilateral hilar lymphadenopathy. A transbronchial lymph node fine-needle biopsy revealed noncaseating granulomas. Considering this finding, probable neurosarcoidosis was diagnosed.

Leptomeningeal involvement is a remarkable finding in neurosarcoidosis. 1 When central nervous system (CNS) involvement occurs concomitantly with a widespread leptomeningeal enhancement, sarcoidosis should be considered as a possible differential diagnosis. 2
